# Moderate Exercise Training Combined With a High-Fat and Sucrose Diet Protects Pancreatic Islet Function in Male C57BL/6J Mice

**DOI:** 10.3389/fendo.2022.881236

**Published:** 2022-05-20

**Authors:** Katherine Veras, Camila Ferraz Lucena, Julia Goedcke, Fabiana S. Evangelista, Angelo Carpinelli, Carla Roberta de Oliveira Carvalho

**Affiliations:** ^1^Institute of Biomedical Sciences, Department of Physiology and Biophysics, University of São Paulo, São Paulo, Brazil; ^2^Non-Communicable Diseases Research Unit, South African Medical Research Council, Cape Town, South Africa; ^3^School of Arts, Sciences and Humanities, University of São Paulo, São Paulo, Brazil

**Keywords:** high fat and sucrose diet, aerobic exercise training, obesity, pancreatic islets, glucose tolerance, insulin sensitivity

## Abstract

Obesity is mainly caused by excess energy intake and physical inactivity, and the number of overweight/obese individuals has been steadily increasing for decades. Previous studies showed that rodents fed westernized diets exhibit endocrine pancreas deterioration and a range of metabolic disorders. This study evaluated the effects of moderated aerobic treadmill exercise training on pancreatic islet cell viability and function in mice consuming a high-fat and sucrose diet. In the present study, 60-day-old male C57BL/6J mice were divided into four groups: control (C), fed a standard diet AIN-93M (3.83 kcal/g; 70% carbohydrate (cornstarch and dextrinized starch were chosen as the major source of carbohydrate for the AIN-93 diet. In addition, a small amount of sucrose), 20% protein (casein), and 10% fat (soybean) with no training (i.e., sedentary); C + training (CTR, fed the standard diet with eight weeks of exercise; high-fat diet + sucrose (HFDS), fed a high fat and sucrose diet (5.2 kcal/g; 20% carbohydrate (cornstarch and dextrinized starch were chosen as the major source of carbohydrate), 20% protein (casein), 60% fat (Lard was chosen as the major source of fat and a small amount of soybean) + 20% sucrose diluted in drinking water with no training; and HFDS + training (HFDSTR). After eight weeks, the HFDS mice displayed increased body weight (P<0.001) and epididymal, inguinal and retroperitoneal adipose tissue mass (P<0.01). These mice also presented insulin resistance (P<0.01), glucose intolerance (P<0.001), impaired glucose-stimulated insulin secretion (GSIS) and were less responsive to the physiological net ROS production induced by glucose stimulus. The HFDS group’s pancreatic islet cells were 38% less viable and 59% more apoptotic than those from the C group (P<0.05). The HFDSTR improved glucose tolerance, body mass, insulin sensitivity and GSIS (P<0.05). Furthermore, HFDSTR mice had 53% more viable isolated pancreatic islets cells and 29% fewer apoptotic cells than the HFDS group (P<0.01). Thus, exercise training may slow down and/or prevent adverse metabolic effects associated with consuming a westernized diet.

## Introduction

The rising worldwide prevalence of overweight and obese individuals has been described as a global pandemic (1). Notably, no country has reported a decrease in the prevalence or rate of obesity in the last 33 years (2). It is well known that obesity is mostly caused by excess energy intake from diets rich in saturated fat and sugar (i.e., westernized diet) and reduced energy expenditure such as physical inactivity, a combination frequently observed in modern human societies (3–5).

Obesity can lead to insulin resistance (IR) with compensatory insulin secretion to maintain glucose homeostasis (6). Under chronic conditions, this mechanism impairs pancreatic β-cell function, resulting in glucose intolerance and type 2 diabetes mellitus (T2DM) (7). Extensive studies in rodent models have shown that diets resembling those commonly consumed by humans in modernized societies (i.e., westernized diet) cause endocrine pancreas deterioration and metabolic disorders (8, 9). Moreover, a high-fat diet supplemented with 20% sucrose (HFDS) has been utilized in rodents to study obesity-related morbidities (9–11).

The mechanisms whereby a hypercaloric diet can impair the pancreatic endocrine function, including glucose-stimulated insulin secretion (GSIS), may be partially related to decreased cell viability and antioxidant capacity and/or oxidative stress due to unbalanced reactive oxygen species (ROS) production and elimination (12). Indeed, studies have shown that excessive ROS production promotes apoptosis in insulin-secreting cell lines and pancreatic islets from rodents (13, 14). It has also been reported that long-term HFDS feeding induced pancreatic steatosis and increased oxidative stress and β-cell apoptosis in Bama minipigs (15).

Physical exercise promotes several systemic adaptations, including increased insulin sensitivity and improved glucose homeostasis, that reduce obesity and T2DM risks (16, 17). Studies have shown that the insulin response in peripheral tissues to regular exercise may be decreased in healthy individuals because physical activity attenuates the demand on β-cells (18, 19). Exercise also increases cellular antioxidant capacity, shielding β-cells from oxidative stress-induced dysfunction and death (20). Furthermore, exercise protects rodent and human pancreatic β cells under diabetogenic conditions, enhancing β-cell viability (21–23).

Unfortunately, due to the low cost, easy access, and taste, the consumption of highly processed foods high in fat and sugar is increasing globally (24). Previous work showed that combined exercise strategies to improve cardiorespiratory fitness and weight management could prevent diet-induced obesity and associated complications (25, 26). Indeed, a randomized controlled trial showed that two weeks of cycling exercise training improved β-cell function in prediabetic and T2DM individuals and decreased pancreatic fat independent of baseline glucose tolerance (27). Additionally, another study showed that combining moderate running exercise with cola-drinking ameliorated the metabolic disorders and promoted an approximately 50% increase in pancreatic β cell mass observed in the sedentary cola-drinking group (28).

Most of the findings related to this topic are disease models in which the metabolic disorder was already present (23, 29–31). Therefore, our study aimed to investigate the effects of combining aerobic exercise training with a high-fat and sucrose diet on cell viability and β-cell function in pancreatic islets of male C57BL/6J mice. Despite the reduced caloric intake, there was an increased feeding efficiency ratio (FER) and body mass in the HFDS group. These findings provide insights into preventive strategies that could mitigate the deleterious effects of the western diet on the endocrine pancreas and glucose homeostasis.

## Material and Methods

### Ethical Approval

All experiments were performed following the ethical guidelines for experiments with mice. Before starting the study, the protocol was prospectively approved by the Biomedical Sciences Institute’s Animal Health Committee at the University of São Paulo (CEUA). The Brazilian Society of Science in Laboratory Animals (SBCAL) also approved the experimental protocols of this study (protocol #065, page 07 of Book 03).

### Experimental Design

**Figure 1:**
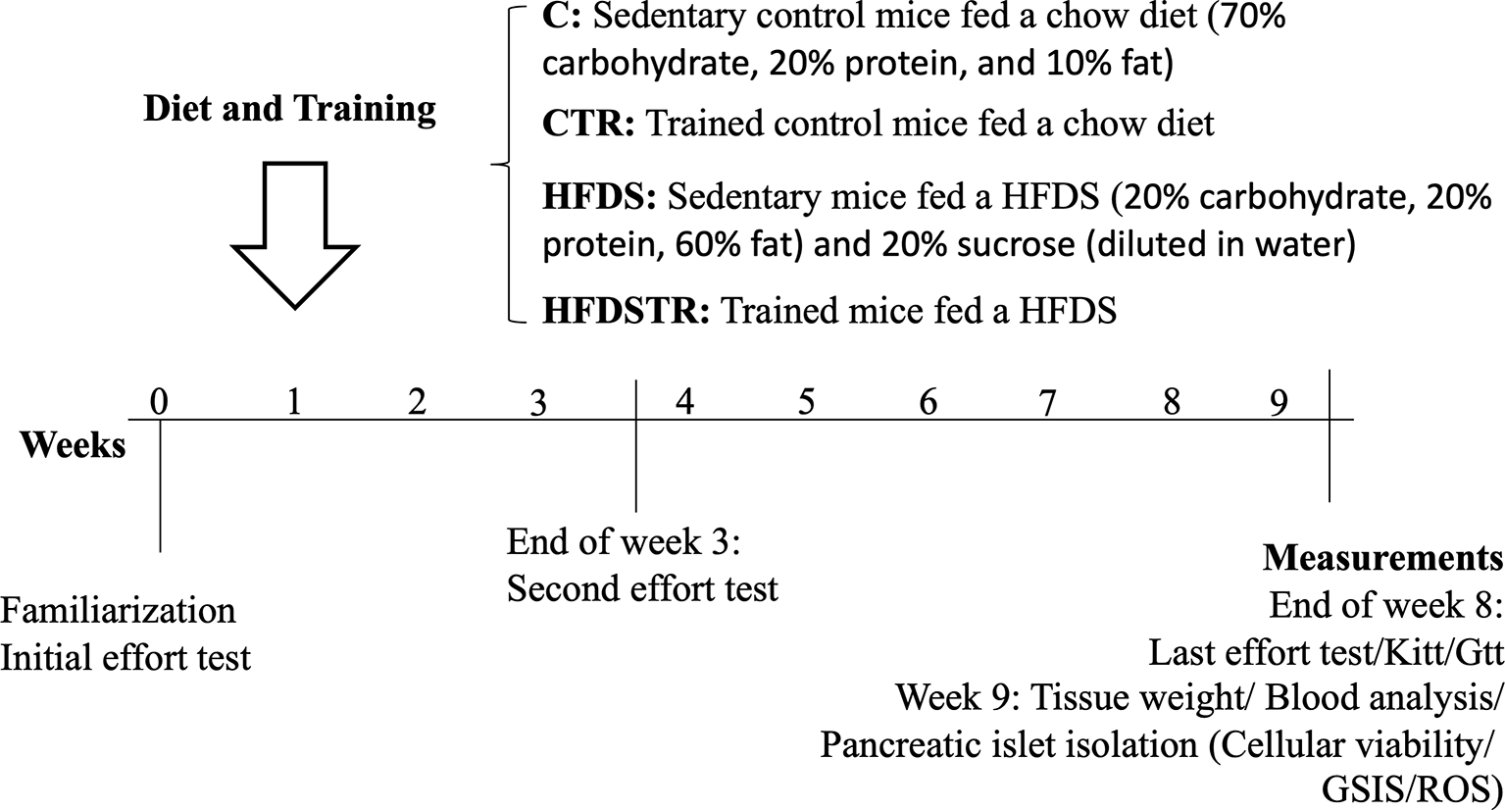
Schematic representation of the experimental design.

Sixty-day-old male C57BL/6J mice were obtained from our breeding colony at the Institute of Biomedical Sciences at the University of São Paulo. These animals were randomly divided into four groups: control (C), fed a standard diet AIN-93M (3.83 kcal/g; 70% carbohydrate (cornstarch and dextrinized starch were chosen as the major source of carbohydrate for the AIN-93 diet. In addition, a small amount of sucrose), 20% protein (casein), and 10% fat (soybean) (32) with no training (i.e., sedentary); C + training (CTR, fed the standard diet with eight weeks of exercise (**Figure 1**); high-fat diet + sucrose (HFDS), fed a high fat and sucrose diet (5.2 kcal/g; 20% carbohydrate (cornstarch and dextrinized starch were chosen as the major source of carbohydrate), 20% protein (casein), 60% fat (Lard was chosen as the major source of fat and a small amount of soybean) + 20% sucrose diluted in drinking water adapted from (32–34) with no training; and HFDS + training (HFDSTR) (**Figure 1**).

The animals were housed in a temperature (22°C ± 2°C) and humidity (60%) controlled room with a 12h light/dark cycle. The mice had free access to food and water throughout the experimental protocol. Body weight and food and water intake were recorded weekly. Total food, water and energy intake were estimated by calculating the area under the curve (AUC) over 8 weeks. The FER was calculated by dividing the weight gained by kilocalories consumed over 8 weeks (35). At the end of the experimental protocol and 48 hours post-exercise training, mice were exposed to 70% CO_2_ and 30% O_2_ and euthanized by decapitation. Tissue weights of the epididymal, inguinal, and retroperitoneal adipose depots and soleus and gastrocnemius skeletal muscles were recorded and normalized to final body weight, and samples were stored at -80°C.

### Exercise Intervention

As described in **Figure 1**, one week before starting the training protocol, mice were familiarized with the treadmill by carrying out four mild exercise sessions at 8.3 m/min for 10 min. The exercise capacity of each animal was evaluated through a maximal effort test (T_max_) on a motorized treadmill, starting at 5 m/min and increasing the speed by 1 m/min until exhaustion. The maximal exercise capacity (run-to-exhaustion) was defined as when the mouse stopped running spontaneously (36, 37). After the initial effort test, mice in the CTR and HFDSTR groups were exercise-trained 30 min/day, five times per week at 60% of the peak velocity treadmill running speed [adapted from (38)]. A T_max_ test was performed at the end of week three to maintain a constant relative intensity throughout the 8-week training period, and the treadmill running speed was adjusted accordingly. Another T_max_ test was conducted after completing the 8-week training period to assess post-test performance changes (**Figure 1**).

### Insulin Sensitivity, Glucose Tolerance and Homeostasis Model Assessments

After eight weeks and 48 hours after the last training session, the animals were restricted from eating for six hours and then subjected to a 30-min insulin tolerance test (short ITT) (n=42) or a glucose tolerance test (GTT) (n=40). For the short ITT, blood glucose was measured (Accue check active, Roche) before delivering (zero time point) an intraperitoneal insulin bolus (0.75 U/Kg) and after 5, 10, 15, 20, 25 and 30 minutes. The glucose disappearance rate (Kitt) was calculated based on the slope from a linear regression of glucose concentration from 5 to 25 minutes after insulin administration (39). For the GTT, blood glucose was measured before an intraperitoneal glucose injection (1 mg/g) (zero time point) and after 15, 30, 45, 60, 90, and 120 minutes. The data were presented as glucose area under the curve (AUC) calculated from 120 min of an intraperitoneal bolus by total area using the trapezoidal rule. The homeostasis model assessment of insulin resistance (HOMA-IR) and the homeostasis model of β-cell function index (HOMA-B) (40) were calculated using fasting glucose and insulin concentrations.

### Fasting Serum Analyses

Forty-eight hours after the last training session, the mice were fasted overnight for 12 hours, decapitated, and then total trunk blood samples were collected (n=101). Serum was separated by centrifugation at 239 × *g* for 20 min at 4°C, and samples were stored at -20°C. Serum insulin concentrations were measured using a radioimmunoassay (RIA; PerkinElmer, Boston, MA, USA) with ^125^I-labeled insulin (41). The serum triglycerides (TG), total cholesterol (TC) and glucose concentrations were determined using an *Accutrend Plus*^®^ device (Roche). The serum aspartate aminotransferase (AST) and alanine aminotransferase (ALT) activities were measured using commercially available kits (Labtest Diagnóstica, Minas Gerais, Brazil). Lipid peroxidation was estimated by quantifying thiobarbituric acid reactive species (TBARS) levels in the serum, as previously described (42).

### Citrate Synthase Activity

Forty-eight hours after the last training session, the maximum citrate synthase (CS) activity was determined in the gastrocnemius muscle (∼20 mg) (n=31). Briefly, 0.5 mM Tris-HCl extraction buffer and 1.0 mM EDTA pH 7.4 was used for protein extraction, and Tris/aminomethane (100 mM), 5,5′-Dithiobis (2-nitrobenzoic acid) (DTNB; 0.2 mM), acetyl CoA (0.1 mM) and Triton X-100 (0.1% v/v), pH 8.1 was used to measure the enzymatic activity. The reaction was initiated by adding 3 μL of muscle homogenate and 10 μL of oxaloacetic acid (10 mM final concentration) to the samples. The reactions were monitored spectrophotometrically by measuring the absorbance at 412 nm at 25°C, as previously described (43). The activity was measured by quantifying the complex formed between Coenzyme A (CoA) released by the medium and oxaloacetate DTNB. The maximal enzyme activity was expressed as nanomoles per minute per milligram of protein.

### Liver Lipid Droplet Determination

The livers of 12 mice were fixed in 10% formaldehyde solution for eight hours in individual cassettes to evaluate liver histology. Then, the fixed samples were incubated in 70% alcohol overnight. Next, the samples were dehydrated through a series of baths in 95% alcohol, 100% alcohol and xylene. Following dehydration, the tissue samples were embedded in paraffin at 60°C, and a microtome (Zeiss, Jena, Germany) cut the samples into 5-micron semi-serial slices, which were then stained with hematoxylin and eosin (H&E). The cellular histology was evaluated, and the intracellular liver lipid droplets (LLDs) were quantified. Twenty images from each animal were obtained using a Nikon Eclipse Ti-U microscope at 20× magnification coupled with a Nikon DS-R1 digital camera and NIS-Elements BR 3.1 software. These images were projected onto a high-resolution LCD monitor. The test system contained 270 symmetrically distributed points produced from a transparent sheet placed on the monitor screen. Each point that remained on the LLDs was counted and named EP. Then the EP/P ratio, which corresponds to the amount of LLDs, was calculated (44). The results were expressed as a percentage.

### Pancreatic Islet Isolation

Forty-eight hours after the last training session and following decapitation, the pancreas from 101 mice were collected, and islets were isolated by collagenase digestion (45, 46). Briefly, the pancreas was inflated with Hanks solution containing 0.68 g/L type IV collagenase (Sigma–Aldrich Chemical, St. Louis, MO, USA), excised and maintained at 37°C for 25 min. The digested tissue was harvested, and the islets were hand-picked. Krebs-Henseleit buffer containing 136 mM NaCl, 5.36 mM KCl, 4.17 mM NaHCO_3_, 1.26 mM CaCl_2._2H_2_O, 0.81 mM MgSO_4_.7H_2_O, 0.3 mM NaHPO_4_.12H_2_O and 0.44 mM KH_2_PO_4_ was used for islet isolation and pooling. The pancreatic islet samples were utilized in the cell viability, DNA fragmentation, GSIS and ROS assays.

### Pancreatic Islet Cellular Viability

Pancreatic islets were disrupted by trypsin digestion and gentle pipetting (47). Living, apoptotic and dead cells were identified with the Guava ViaCountR assay (Millipore, Billerica, MA, USA) based on staining the cytoplasm and nucleus with different dyes. One dye is membrane-permeable and stains all nucleated cells, whereas the other is membrane-impermeable and only stains damaged cells, indicating apoptotic and dying cells. The cell suspensions were incubated with the ViaCount reagent for five minutes at room temperature. Data acquisition was performed on a Guava EasyCyte flow cytometer (Millipore, Billerica, MA, USA), and data analysis was performed using the ViaCount software module.

### Islet Cell Apoptosis

Islet dispersed cell apoptosis detection was performed by using the Cell Death Detection ELISA^PLUS^ kit (Roche Diagnostics, Mannheim, Germany), according to the manufacturer’s instructions. This assay measures the amount of histone-associated DNA fragments (mono- and oligonucleosomes). Herein, batches containing 80 islets per mouse were lysed in 100 μL of lysis buffer, and the cytosolic and nuclear fractions were separated by centrifugation at 200 × *g* for 10 min. The mean absorbance at 405 nm of each sample was normalized to the DNA content in the nuclear fraction.

### Glucose Stimulated Insulin Secretion (GSIS) and Net Cytosolic ROS Production

GSIS was assessed by incubating five islets in 0.5 mL Krebs–Henseleit buffer containing 0.2% albumin and 5.6 mM glucose (pre-incubation phase) at 30°C for 30 min. After the pre-incubation phase, the islets were incubated in 2.8 mM and 16.7 mM glucose for 60 min (47). Then, 300 μL of the supernatant were stored at -20°C until determining “insulin secretion”. For total “insulin content”, the pancreatic islets were disrupted in a 52 ethanol:17 water:1 hydrochloric acid solution and sonicated. The “insulin secretion” and “insulin content” were measured by radioimmunoassay (RIA) (41). The insulin secretion data (corresponding to groups of five islets per glucose concentration) was expressed as the insulin secretion/insulin content ratio.

Islet superoxide content was evaluated by flow cytometry (Guava EasyCyte, Millipore), monitoring its reaction with dihydroethidium (DHE; Life Technologies, Eugene, Oregon, USA) (48), which generates the fluorescent product 2-hydroxyethidium (2-OH-E^+^) (49). Groups of 25 islets were pre-incubated at 37°C for 30 min in Krebs-Henseleit buffer, pH 7.4 containing 5.6 mM glucose, and then incubated for one hour in Krebs-Henseleit buffer with 0.2% BSA and 2.8 mM or 16.7 mM glucose (47). Next, 50 µL DHE was added, and the samples were incubated in the dark at room temperature for 20 min. The islets were trypsin-digested, gently pipetted up and down and resuspended in 200 µL of RPMI 1640 culture medium at 37°C for two minutes. Afterward, an additional 600 µL of RPMI-1640 medium was added to inactivate the trypsin, as described previously with adaptations (47). The yellow fluorescence was measured using an excitation wavelength of 488 nm, and the emission signal was acquired with a yellow filter (583 nm ± 26 nm). Despite the superoxide radical’s short half-life, 5000 events were recorded per group of 25 islets per mouse.

### Statistical Analysis

GraphPad Prism v.9 (GraphPad Software, La Jolla, CA, USA) was used to perform the statistical analyses. All data are expressed as the mean ± standard deviation (SD) using a scatter dot plot that allows the reader to visualize the number of individual samples in each analysis. Two-way ANOVA with Tukey’s *post-hoc* test was used to analyze diet and training effects. For all analyses, a P-value of <0.05 was considered statistically significant.

## Results

### Exercise Training Efficiency

**Figure 2:**
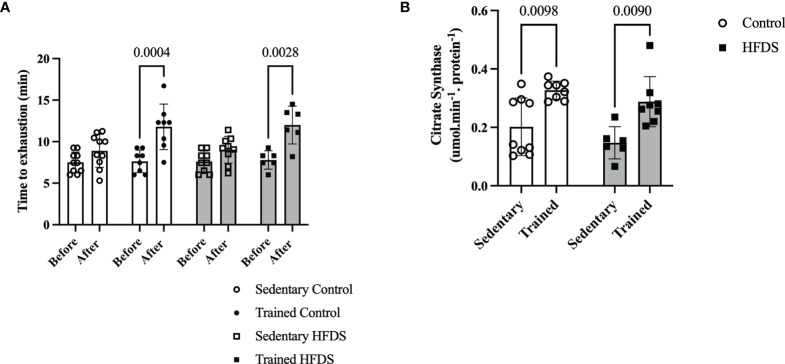
Effects of endurance training on exercise capacity (run-to-exhaustion time). **(A)** C, CTR, HFDS and HFDSTR (week 0 *vs*. after week 8). Differences in CTR and HFDTR between week 0 *vs*. 8, respectively (P<0.01). **(B)** Citrate Synthase Activity in the gastrocnemius muscle of C, CTR, HFDS and HFDSTR after eight weeks: Endurance training effect (P<0.01). Data are expressed as the mean ± SD.

Exercise training, irrespective of the diet, resulted in significant increases in run-to-exhaustion times [F= (1, 58) = 39.78, P<0.0001] (**Figure 2A**). The run-to-exhaustion times of the CTR and HFDSTR groups increased by 55.3 and 53.8%, respectively (CTR, week 8 *vs*. 0: -4.155, 95% CI -6.932 to -1.378, P=0.0004 and HFDS, week 8 *vs*. 0: -4.208, 95% CI -7.415 to -1.002, P = 0.0028). On the other hand, the C (-1.395, 95% CI -3.879 to 1.089, P = 0.6448) and HFDS (-1.413, 95% CI -4.031 to 1.205, P = 0.6888) groups were not significantly different (18.7 and 18.4%, respectively) (**Figure 2A**). As shown in **Figure 2B**, CS activity in the gastrocnemius muscle increased by 1.6 and 2-fold in the exercise-trained groups compared to their respective controls without training (CTR *vs.* C: -0.1251, 95% CI -0.2247 to -0.02541, P = 0.0098) and HFDSTR *vs.* HFDS: -0.1405, 95% CI -0.2513 to -0.02971, P = 0.0090).

### Body Weight Changes

**Figure 3:**
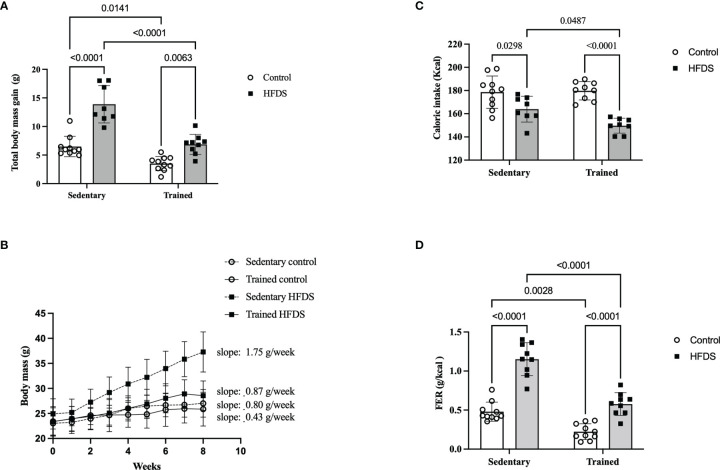
Effects of eight weeks of exercise training and HFDS diet on metabolic parameters in male C57BL6J mice. **(A)** Total body mass gain after eight weeks. C *vs*. HFDS, HFDS *vs*. HFDSTR, CTR *vs*. HFDSTR (P<0.01). **(B)** Changes in body mass over the eight-week intervention. C *vs*. CTR (P<0.05), C *vs*. HFD (P<0.001), HFDS *vs*. HFDSTR and CTR *vs*. HFDSTR (P<0.05). **(C)** Total caloric intake; C *vs*. HFDS, HFDS *vs*. HFDSTR (P<0.01). **(D)** FER: C *vs*. CTR, C *vs*. HFDS, HFDS *vs*. HFDSTR and CTR *vs*. HFDSTR (P<0.001). Data were analyzed by two-way ANOVA as described in the Materials and Methods. All data presented as the mean ± SD.

The CTR and HFDS animals displayed a 1.8-fold-decrease and 2.1-fold increase, respectively (3.5 ± 1.2g and 14 ± 3.2g) in total body mass gain compared to the C mice [C *vs*. CTR: 3.0, 95% CI 0.5 to 5.50, P<0.01; C *vs*. HFDS: -7.4, 95% CI -10 to -4.7, P<0.0001; HFDS *vs*. HFDSTR: 7.01, 95% CI 4.3 to 9.74, P<0.0001)] (**Figure 3A**). Furthermore, the HFDSTR mice had 2-fold decreased the total body mass gain than in HFDS group (HFDS *vs* HFDSTR: 7.01, 95% CI 4.3 to 9.74, P < 0.0001). Throughout the eight-week experimental period, the CTR mice gained 0.43 g of b.w./week while the HFDS mice gained 1.75 g/week on average compared to the C and HFDSTR groups which only gained 0.80 and 0.87 g/week (P<0.0001, **Figure 3B**).

### Food Intake

As shown in **Figure 3C**, the caloric intake was reduced in both HFDS and HFDSTR by 90 and 85% compared to the C and CTR groups, respectively (C *vs*. HFDS: 14.60, 95% CI 0.8319 to 28.37, P=0.0343 and CTR *vs*. HFDSTR: 27.30, 95% CI 13.96 to 40.64, P<0.0001). Nonetheless, the FER was decreased in CTR mice compared to the C group (C *vs*. CTR: 0.2556, 95% CI 0.07577 to 0.4355, P=0.0028) (**Figure 3D**). Despite the 14 kcal decrease in the HFDS animals’ total caloric intake during the eight-week experiment (C *vs*. HFDS: -0.6730, 95% CI -0.8578 to -0.4882, P<0.0001) (**Figure 3C**), there was an increase in the FER, an observation that was attenuated in the HFDSTR mice (HFDS *vs*. HFDSTR: 0.5750, 95% CI 0.3854 to 0.7646, P<0.0001) (**Figure 3D**).

### Tissue Weights and Liver Lipid Droplets Percentages

**Table 1** shows the Tissue Weights. Despite adjusting for body weight, a diet effect (P<0.05) was observed and the HFDS group presented increased epididymal (C *vs*. HFDS: -2.292, 95% CI -3.487 to -1.097, P<0.0001) inguinal (C *vs*. HFDS: -1.792, 95% CI -3.349 to -0.2341, P=0.0191) and retroperitoneal (C *vs*. HFDS: -1.664, 95% CI -2.546 to -0.7828, P<0.0001) WAT weights, while the soleus [F (1, 28) = 0.01299, P=0.9101] and gastrocnemius [F (1, 32) = 0.06137, P=0.8059] muscle weights were not significantly altered. Additionally, exercise training did not alter the weights of the epididymal [F (1, 53) = 6.106, P=0.0167], inguinal [F (1, 32) = 1.933, P=0.1740] and retroperitoneal [F (1, 31) = 2.508, P=0.1234] tissues or the soleus [F (1, 28) = 3.753, P=0.0629] and gastrocnemius [F (1, 32) = 0.06137, P=0.8059] muscles (**Table 1**).

As expected, there were no detectable (ND) LLDs in the hepatocytes of the C and CTR mice (0.000, 95% CI -6.316 to 6.316, P>0.9999). In contrast, the LLD percentage in the HFDS mice was markedly increased to over 35% (-37.75, 95% CI -44.45 to -31.05, P<0.0001). Notably, the hepatocytes from the HFDSTR group contained 39.5% fewer liver LLDs than those from HFDS mice (15.25, 95% CI 9.134 to 21.37, P<0.0001) (**Table 1**).

**Table 1:** Tissue weights and LLDs in hepatocytes following the eight-week intervention in the C, CTR, HFDS and HFDSTR groups.

Variables	N	C	CTR	HFDS	HFDSTR
**Epididymal WAT**	47	2.15 ± 0.97	1.67 ± 0.96	4.43 ± 1.63*	3.24 ± 1.43^#^
**Inguinal WAT**	36	1.72 ± 0.80	1.53 ± 0.88	3.51 ± 1.81*	2.53 ± 1.28
**Retroperitoneal WAT**	35	1.07 ± 0.43	0.86 ± 0.38	2.73 ± 1.02*	2.21 ± 0.72
**Soleus Muscle**	32	0.07 ± 0.007	0.07 ± 0.007	0.06 ± 0.02	0.08 ± 0.02
**Gastrocnemius Muscle**	36	0.83 ± 0.31	1.0 ± 0.16	0.81 ± 0.10	0.97 ± 0.15
**LLDs**	12	ND	ND	37.2 ± 2.5*	22.5 ± 5.4^#$^

Tissues weights are expressed as g/g body mass. LLDs were expressed as a percentage. N indicates the total number of mice used. ND is non-detectable. Symbols represents pos-hoc test (P<0.05): *C vs. HFDS and ^#^CTR vs. HFDSTR and ^$^HFDS vs. HFDSTR. Data are expressed as the mean ± SD.

### Biochemical Analysis

A summary of the biochemical marker data for each group is presented in **Table 2**. Compared to C, the HFDS group presented higher glucose (-73.98, 95% CI -94.89 to -53.07, P<0.0001), insulin (0.3367, 95% CI 0.07933 to 0.5940, P=0.0068), HOMA-B (-23.54, 95% CI -42.39 to -4.694, P<0.005) and TG (22.02, 95% CI 1.838 to 42.19, P<0.005). Several of these variables including glucose, (61.33, 95% CI 40.69 to 81.96, P<0.0001), insulin (-0.3492, 95% CI -0.6065 to -0.09183, P=0.0049), and HOMA-B (38.70, 95% CI 18.83 to 58.57, P<0.0001) were decreased in the HFDSTR group. There were no significant alterations in HOMA-IR [(F (1, 27) = 0.03797, P=0.8470], TC values [F (1, 30) = 0.8323, P=0.3689], ALT [F (1, 14) = 0.1409, P=0.7130] and AST [F (1, 14) = 8.490e-9, P>0.9999] activities, or TBARS levels [F (1, 28) = 0.1480, P=0.7033] between groups.

**Table 2:** Serum biochemical profile following the eight-week intervention in the C, CTR, HFDS and HFDSTR groups.

Variables	N	C	CTR	HFDS	HFDSTR
**Insulin (ng/dL)**	31	0.71 ± 0.12	0.75 ± 0.09	1.05 ± 0.02*	0.7 ± 0.18^$^
**Glucose (mg/dL)**	15	171.30 ± 29.63	162.5 ± 19.46	245.3 ± 32.72*	184.0 ± 18.16^$^
**HOMA-IR (UA)**	31	2.52 ± 0.18	2.60 ± 0.19	2.80 ± 0.23	2.85 ± 0.30
**HOMA-β (UA)**	21	67.70 ± 7.12	54.4 ± 11.81	90.3 ± 3.17*	51.6 ± 12.26^$^
**TG (mg/dL)**	32	118.42 ± 12.43	111.80 ± 6.50	140.40 ± 13.23*	154.12 ± 22.02^#^
**TC (mg/dL)**	31	175.75 ± 3.65	173.66 ± 3.77	176.62 ± 2.38	170.25 ± 6.75
**ALT (UI/mL)**	18	1.10 ± 0.09	1.10 ± 0.07	1.40 ± 0.28	1.32 ± 0.18
**AST (UI/mL)**	18	1.10 ± 0.11	1.10 ± 0.06	1.25 ± 0.20	1.25 ± 0.17
**AST/ALT**	18	1.01 ± 0.05	1.00 ± 0.01	1.11 ± 0.13	1.05 ± 0.01
**TBARS (nmol MDA/mL)**	32	0.70 ± 0.17	0.63 ± 0.30	0.58 ± 0.40	0.46 ± 0.21

TG, triglycerides; TC, total cholesterol. Differences (P<0.05) were observed in *C vs. HFDS and ^#^HFDS vs. HFDSTR, and ^$^HFDS vs. HFDSTR. Data are expressed as the mean ± SD.

### Insulin Sensitivity and Glucose Tolerance

**Figure 4:**
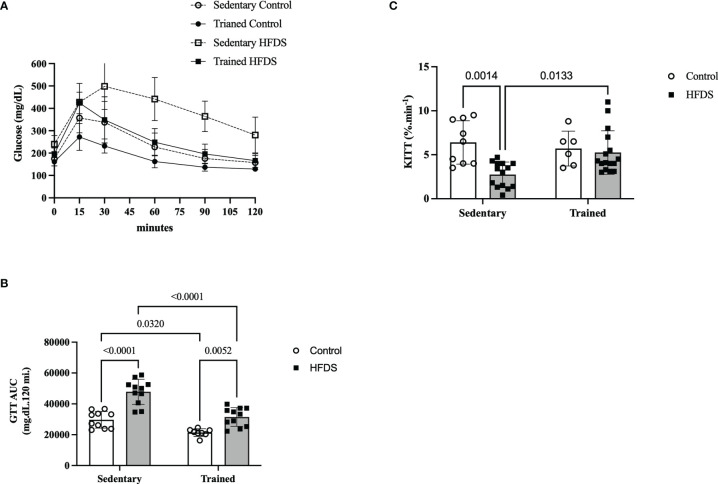
Glucose and insulin sensitivity test following the eight-week intervention in the C, CTR, HFDS and HFDSTR groups. Data were analyzed by two-way ANOVA as described in the Material and Methods. **(A)** GTT represented by **(B)** AUC: C *vs.* CTR; C vs. HFDS; HFDS *vs*. HFDSTR and CTR *vs.* HFDSTR (P<0.05). **(C)** Kitt: C *vs*. HFDS and HFDS *vs.* HFDSTR (P<0.01). Data are expressed as the mean ± SD.

The GTT of HFDS mice (**Figure 4A**), also represented by the AUC of glucose in **Figure 4B**, was 61% greater than in the C group (18102, 95% CI -25249 to -10956, P<0.0001) and 55% greater than the HFDSTR group (16291, 95% CI 9317 to 23265, P<0.0001). The AUCs of glucose for the C and HFDSTR groups were not significantly different (-1812, 95% CI -8958 to 5334, P=0.9030). In contrast, the Kitt of the HFDS group was 2.3-fold lower than the C group (3.657, 95% CI 1.214 to 6.100, P=0.0014). Furthermore, as shown in **Figure 4C**, the Kitt of the HFDSTR mice was 1.9-fold higher than that of the HFDS mice (-2.507, 95% CI -4.600 to -0.4147, P=0.0133) but was not significantly different from the C group (-1.232, 95% CI to 3.532, P = 0.5729).

### Pancreatic Islet Cellular Viability and Islet Cell Apoptosis

**Figure 5:**
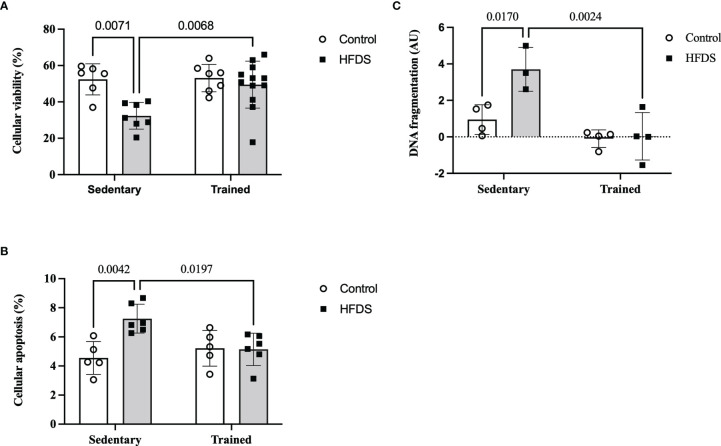
Dispersed pancreatic islet after the eight-week intervention in the C, CTR, HFDS and HFDSTR groups. **(A)** Percentage of viable pancreatic cells: C *vs.* HFDS and HFDS *vs*. HFDSTR (P<0.05). **(B)** Cellular apoptosis: C *vs.* HFDS and HFDS vs. HFDSTR (P<0.05). **(C)** DNA fragmentation: C *vs.* HFDS and HFDS *vs*. HFDSTR (P<0.05). Data are expressed as the mean ± SD.

As expected, the cellular viability of dispersed pancreatic islets was reduced by 38% in the HFDS group compared to the C group (19.97, 95% CI 4.615 to 35.32, P=0.0071). This effect was attenuated in the HFDSTR mice (-17.17, 95% CI -30.29 to -4.040, P=0.0068) which presented 53% more viable cells *vs* HFDS (**Figure 5A**). Conversely, cellular apoptosis (**Figure 5B**) and DNA fragmentation (**Figure 5C**) increased 1.6 (-2.7, 95% CI -4.605 to -0.7947, P=0.0042) and 3.9-fold (2.752, 95% CI -5.018 to -0.4859, P=0.0170) in the HFDS mice compared to the C group. Notably, in the HFDSTR mice, there was a 29% reduction in cellular apoptosis (2.108, 95% CI 0.2920 to 3.925 P=0.0197), accompanied by a 99% reduction in the DNA fragmentation compared to the HFDS group (3.669, 95% CI 1.403 to 5.935, P=0.0024), and not significantly different from C mice (-0.5913, 95% CI -2.496 to 1.314, P = 0.8165) and (0.9167, 95% CI -1.181 to 3.015, P = 0.5729), respectively.

### Glucose Stimulated Insulin Secretion (GSIS) and Net Cytosolic ROS Production

**Figure 6:**
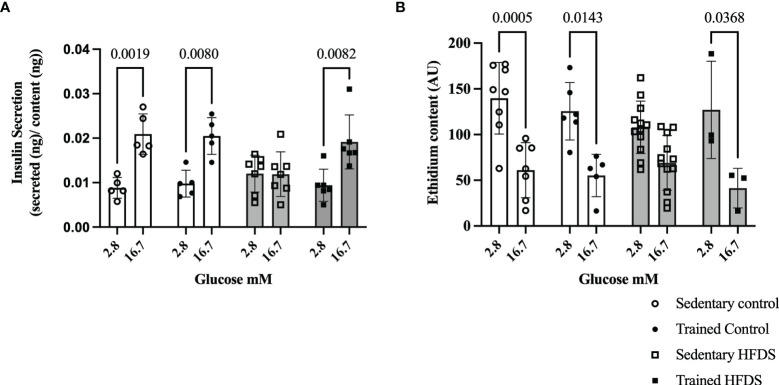
Effects of eight weeks of exercise training and HFDS diet on pancreatic islets. **(A)** GSIS normalized for total insulin content. Glucose responsiveness (2.8 *vs*. 16.7mM) in C (P<0.0001), CTR (P<0.0001) and HFDSTR groups (P<0.0001). **(B)** Ethidium bromide content and average fluorescence intensity by DHE in dispersed pancreatic islets after 1-hour of glucose stimulus (2.8 vs. 16.7mM). Glucose effect (2.8 *vs*. 16.7mM) in C, CTR and HFDSTR (P<0.001). Data are expressed as the mean ± SD.

The GSIS rate was increased 2-fold with high glucose (16.7 mmol/L) compared to low glucose (2.8 mmol/L) in the C (-0.01210, 95% CI -0.02088 to -0.003325, P=0.0019), CTR (-0.01071, 95% CI -0.01949 to -0.001934, P=0.0080) and HFDSTR (-0.009751, 95% CI -0.01776 to -0.001739, P=0.0082) groups. As expected, the HFDS group did not display an increase in static insulin secretion induced by the high glucose concentration (0.0001243, 95% CI -0.007293 to 0.007542, P>0.9999) (**Figure 6A**). Lastly, reduced indirect ROS levels were detected in the high glucose condition compared to the low glucose in the C (78.58, 95% CI 26.26 to 130.9, P=0.0005), CTR (70.29, 95% CI 9.079 to 131.5, P=0.0143) and HFDSTR (85.68, 95% CI 3.135 to 168.2, P=0.0368) groups, but not in the HFDS mice (39.00, 95% CI -2.267 to 80.27, P=0.0764) (**Figure 6B**).

## Discussion

The present study showed that moderate-intensity exercise training could avoid some of the deleterious effects of a high fat and sucrose diet on murine pancreatic cell viability, glucose tolerance and insulin sensitivity. The benefits of this specific physical exercise regimen were associated with maintaining GSIS and cytosolic ROS levels, consequently improving glucose tolerance and insulin sensitivity.

Despite the HFDS group ingesting fewer calories, the FER was increased, and these animals gained more body and WAT weight than the C group. A classical situation of body mass gain, particularly fatty mass, is related to reduced energetic expenditure and increased energetic intake. Unfortunately, in our study, we could not analyze indirect calorimetry. However, evidence indicates that long-term ingestion of a sugar-rich or high-fat diet causes severe obesity in rats without hyperphagia (50). On the other hand, another study (51) showed that energy intake was similar between the high-fat and low-fat diet groups due to the HFD group consuming less food. The latter study also showed that energy expenditure was lower in the HFD group than in the low-fat diet group, increasing body weight and fat mass and contributing to insulin resistance. Accordingly, the HFDS mice presented impaired glucose tolerance, higher body fat mass, reduced caloric intake, and insulin sensitivity in the present study.

We found that exercise (CTR *vs*. C and HFDSTR *vs*. HFDS) attenuated the observed weight gain and elevated the FER but did not affect the epididymal, inguinal, and retroperitoneal tissue weights. Notably, the average weight of the HFDSTR mice was comparable to the C group. It is plausible that intramuscular and other ectopic fat content could be involved in body weight regulation, insulin sensitivity and glucose tolerance and improved by exercise training (52). Future studies measuring the whole fat and fat-free mass content are needed to investigate this possibility.

The HFDS group displayed increased TG levels and a higher percentage of LLDs than the C group, but the AST and ALT activities remained unchanged. Non-alcoholic fatty liver disease (NAFLD) is characterized by the aberrant intracellular accumulation of lipids (>5%) in hepatocytes (53) and is highly correlated with obesity, insulin resistance and T2DM (54). While exercise training did not change the TG content in HFDSTR mice, it attenuated the hepatic steatosis grade in these animals. In this sense, exercise could delay non-alcoholic steatohepatitis progression.

We also evaluated endocrine pancreas adaptations by monitoring insulin and glucose metabolism in response to a high-fat and sucrose diet and exercise training. The HFDS mice had fewer viable pancreatic islets, impaired GSIS, reduced cytosolic ROS content, and elevated serum insulin concentrations and HOMA-β values, likely due to the excess intake of fatty acids and sugar. Indeed, previous studies have reported that increased β-cell mass over time may lead to a reduced β-cell mass, culminating in β-cell failure (9, 55). Additional studies evaluating the parameters involved in the pancreatic β-cell adaptations are necessary to elucidate further the precise mechanisms involved.

ROS are constitutively produced and removed, driving a redox state and acting as a signal for cell processes, including pancreatic β-cell signaling and control of insulin secretion (56). Physiologically, acutely elevated glucose concentrations reduce net ROS detection, preventing pancreatic cells from these powerful free radicals and oxidants (57). It is important to point out that the redox homeostasis systems of eukaryotic cells are highly compartmentalized (58), and we only measured net cytosolic ROS production and not mitochondrial and non-mitochondrial ROS production and activity. Our findings demonstrated that the HFDS mice lost, at least in part, the GSIS-associated mechanism with no significant alterations in the systemic TBARS levels compared to the C group. However, the pancreatic islets from HFDS fed mice were less responsive to the physiological net ROS production induced by glucose stimulus. Thus, our results indicate that moderate exercise training effectively stimulates protective hormetic responses (59) and ROS maintenance (60) to compensate for the peripheral insulin resistance elicited by the high-fat and sucrose diet.

Once again, we did not measure antioxidant enzyme content, which could be associated with the observed reduction in ROS production and apoptotic protein content in the pancreatic islet of rats after eight weeks of endurance training (60, 61). Importantly, the eight-week exercise training protocol used in the present study attenuated and prevented the high-fat and sucrose diet-induced effects in mice, evidenced by the maintenance of glucose homeostasis and insulin sensitivity in the HFDSTR animals. These findings are probably associated with more efficient pancreatic islets since exercise increased cell viability and reduced DNA fragmentation and apoptosis, maintaining ROS net content, and preventing GSIS impairment.

Although these results are not necessarily surprising, our study provides valuable insights into how hypercaloric diets and regular aerobic exercise training could prevent or improve obesity-related complications. However, it is unclear if exercise alone can offset the negative effects of foods rich in fat and sugar. Additional studies are required to explore the molecular mechanisms underlying the observed responses and adaptations.

## Conclusion

Consistent with the hypothesis of this study, we showed that moderate exercise training performed simultaneously with a hypercaloric diet protected insulin sensitivity, glucose tolerance and pancreatic islets function in male C57BL/6J mice. Furthermore, this study provides experimental evidence demonstrating that moderate aerobic exercise training could attenuate the risks and complications associated with the westernized diet.

## Data Availability Statement

The raw data supporting the conclusions of this article will be made available by the authors, without undue reservation.

## Ethics Statement

The animal study was reviewed and approved by Biomedical Sciences Institute’s Animal Health Committee at the University of São Paulo (CEUA). The Brazilian Society of Science in Laboratory Animals (SBCAL) also approved the experimental protocols of this study. The approved protocol number is 065, as stated on Sheet 07 of Book 03.

## Author Contributions

KV contributed to the project’s conception and experimental design, data collection, analysis, and interpretation, and drafting of and revising of the article. CM and FE performed experiments and collected data. JG interpreted the data and revised the manuscript. AC and CCRO conceived and designed the experiments, analyzed, and interpreted the data and interpretation and revised the manuscript. All authors contributed to the article and approved the submitted version.

## Funding

This work was supported by Conselho Nacional de Desenvolvimento Científico e Tecnológico (CNPq number: 159288/2011-8) and Fundação de Apoio à Pesquisa do Estado de São Paulo (FAPESP Number: 2019/20464-8).

## Conflict of Interest

The authors declare that the research was conducted in the absence of any commercial or financial relationships that could be construed as a potential conflict of interest.

## Publisher’s Note

All claims expressed in this article are solely those of the authors and do not necessarily represent those of their affiliated organizations, or those of the publisher, the editors and the reviewers. Any product that may be evaluated in this article, or claim that may be made by its manufacturer, is not guaranteed or endorsed by the publisher.
